# Membrane-Based Technologies for Post-Combustion CO_2_ Capture from Flue Gases: Recent Progress in Commonly Employed Membrane Materials

**DOI:** 10.3390/membranes13120898

**Published:** 2023-12-02

**Authors:** Petros Gkotsis, Efrosini Peleka, Anastasios Zouboulis

**Affiliations:** Laboratory of Chemical and Environmental Technology, Department of Chemistry, Faculty of Sciences, Aristotle University, GR-54124 Thessaloniki, Greece; petgk@chem.auth.gr (P.G.); peleka@chem.auth.gr (E.P.)

**Keywords:** greenhouse gases (GHG), post-combustion CO_2_ capture, membrane-based technologies, gas permeation, membrane materials, composite membranes, mixed-matrix membranes

## Abstract

Carbon dioxide (CO_2_), which results from fossil fuel combustion and industrial processes, accounts for a substantial part of the total anthropogenic greenhouse gases (GHGs). As a result, several carbon capture, utilization and storage (CCUS) technologies have been developed during the last decade. Chemical absorption, adsorption, cryogenic separation and membrane separation are the most widely used post-combustion CO_2_ capture technologies. This study reviews post-combustion CO_2_ capture technologies and the latest progress in membrane processes for CO_2_ separation. More specifically, the objective of the present work is to present the state of the art of membrane-based technologies for CO_2_ capture from flue gases and focuses mainly on recent advancements in commonly employed membrane materials. These materials are utilized for the fabrication and application of novel composite membranes or mixed-matrix membranes (MMMs), which present improved intrinsic and surface characteristics and, thus, can achieve high selectivity and permeability. Recent progress is described regarding the utilization of metal–organic frameworks (MOFs), carbon molecular sieves (CMSs), nanocomposite membranes, ionic liquid (IL)-based membranes and facilitated transport membranes (FTMs), which comprise MMMs. The most significant challenges and future prospects of implementing membrane technologies for CO_2_ capture are also presented.

## 1. Introduction

Global warming, which is caused by the increase in greenhouse gases (GHGs) in the atmosphere, has become a considerable concern as it poses a major threat to human health, energy security and ecosystems [1]. Carbon dioxide (CO_2_) emissions, which result from the combustion of fossil fuels and from industrial processes, account for approximately 65% of the total anthropogenic GHGs globally [2]. Almost 40% of these emissions result from the electricity production of coal-fired power plants [3]. As a result, carbon (CO_2_) capture, utilization and storage (CCUS) technologies are urgently required to minimize CO_2_ emissions and maintain climate temperature [4,5].

Carbon capture processes are categorized into four main groups: (i) post-combustion capture, (ii) pre-combustion capture, (iii) oxy-fuel combustion capture and (iv) capture from industrial process streams. The selection of the applied process depends on the gas stream composition and the emission site; in some cases, CO_2_ capture is required at the beginning of the process operation, while others may require CO_2_ capture at the end of the process operation (exhaust phase) [6]. Post-combustion capture is the separation of CO_2_ from flue gases, which are produced after the combustion of fossil fuels or biomass and generally contain 73–77% N_2_, 15–16% CO_2_, H_2_O (5–7%), O_2_ (3–4%) and other impurities, such as SO_x_ and NO_x_ [3]. Post-combustion capture process is regarded as a technically and economically viable solution for reducing carbon emissions in a variety of sectors/emitters, where decarbonization is possible, but costly in the near future. These emitters include fundamental industrial sectors, which rely on the combustion of fossil fuels, such as power generation plants and steel or cement production industries, but also secondary emitters, such as chemical plants and waste incinerators. Compared to the other three carbon capture processes, post-combustion capture can be easily retrofitted to existing plants and, therefore, it may be the only effective and economic way to reduce CO_2_ emissions without affecting the process upstream, leading to the transition towards net-zero industries [7,8].

The most common post-combustion CO_2_ capture technologies include chemical absorption (with liquid solvents), adsorption (with solid adsorbents), cryogenic separation and membrane separation. Among them, chemical absorption is the most mature technology as it can achieve high CO_2_ capture capacity and removal efficiency, reaching 100% when monoethanolamine (MEA) is employed as a solvent. However, chemical absorption with amines presents several challenges, such as high requirements for energy regeneration, a low reaction rate and a strong environmental impact due to the presence of corrosive or volatile solvents. As a result, alternative carbon capture technologies are increasingly examined both on a research level (lab-scale applications) and in real field conditions (pilot- or full-scale plants) [9].

Unlike CO_2_ capture technologies with solvents or adsorbents, CO_2_ capture with membranes presents significant benefits, such as small footprint, easy scale-up, low capital and operating cost and low energy consumption, as they can deliver high-pressure CO_2_ without utilizing chemical solutions or energy-intensive steam. Membrane separation technology, which was first developed in the 1980s, employs CO_2_-selective membranes to separate CO_2_ from a gas stream [10,11]. This technology has been applied successfully in other applications as well, such as biogas upgrading and natural gas purification. Despite its advantages, however, the major drawback of membrane separation still remains to be the trade-off between selectivity and permeability, which are the most important factors affecting process efficiency and economics [12,13]. Therefore, the development of novel membrane technologies which capture CO_2_ more efficiently and selectively is required. Over the last few years, several studies reviewed and compared membrane-based technologies for CO_2_ capture from flue gases or other gas streams. Da Conceicao et al. (2023) [14] summarized the literature that is linked to the development or application of membrane separation in terms of separation modeling and process simulations. Singh et al. (2022) [15] featured current advancements in CO_2_ capture with membranes, but focused mainly on the utilization of polymeric membrane materials. In their review on membrane technologies for post-combustion carbon capture, Favre et al. (2022) [16] provided a compact, but rather limited, overview regarding commonly employed membrane materials, as they did not include the latest advancements in composite membranes, which are increasingly fabricated and applied in recent years. The present study aims to review the main post-combustion CO_2_ capture technologies from flue gases, but from the standpoint of the progress which has recently been made after the application of membrane-based technologies. Specifically, the objective of the present review is to present the state of the art in novel membrane materials for carbon capture, and primarily focuses on the utilization of mixed-matrix membranes (MMMs) with enhanced intrinsic and surface properties that promote CO_2_ capture.

## 2. Main Processes for CO_2_ Capture

**Figure 1:**
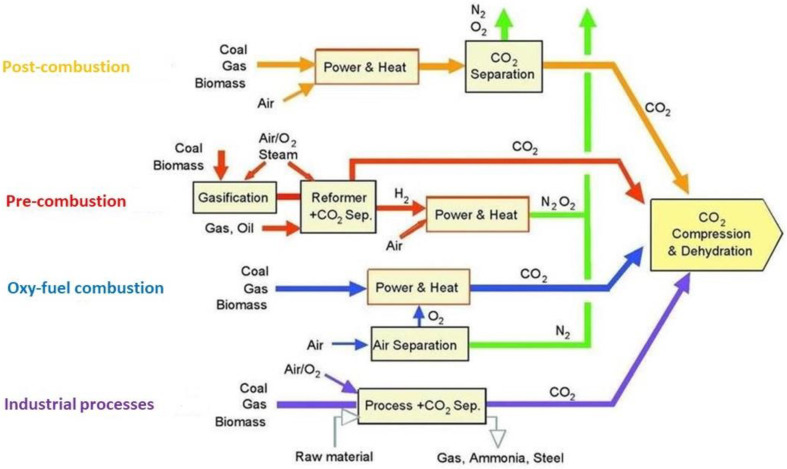
The main CO_2_ capture processes [18,19].

CO_2_ capture from various gas streams can be classified as follows (Figure 1): (i) post-combustion capture, (ii) pre-combustion capture, (iii) oxy-fuel combustion capture and (iv) capture from industrial process streams. Selecting the appropriate capture process depends on the CO_2_ content in the gas stream, the gas stream pressure and the type of fuel used (whether it is solid or gas); thus, not all capture processes are compatible with all systems. All processes, however, involve separating CO_2_, O_2_ or H_2_ from a gas stream, such as a flue gas, air, a natural gas or a biogas, and can be achieved using physical or chemical solvents, solid sorbents, membranes, cryogenic separation or via a combination of these methods [17,18,19,20,21].

Every CO_2_ capture process has advantages and disadvantages. Post-combustion capture allows the easy retrofit of existing plants and, thus, an immediate reduction in emissions. The disadvantages include inefficient capture due to the low partial pressure of CO_2_ and the presence of impurities that degrade the employed solvents. Pre-combustion capture benefits from the high partial pressure of CO_2_ and concentrated gas streams, which results in efficient absorption and reduced solvent consumption. Nevertheless, the main drawback lies in the demand for gasification, which increases process cost and complexity. In oxy-fuel capture, a fuel is burnt with oxygen of high purity, and a concentrated CO_2_ stream that promotes absorption is generated. However, the air separation process, which is required for oxy-fuel combustion, is very energy-intensive [22,23,24].

### 2.1. Post-Combustion Capture

Post-combustion capture refers to the separation of CO_2_ from flue gases, which are produced after the combustion of fossil fuels or biomass, and usually present a low CO_2_ content (3–20%), a low partial pressure of CO_2_ (0.03–0.2 bar) and high temperatures (120–180 °C), but contain NO_x_ and SO_x_ impurities. During this process, the flue gas is not directly discharged into the atmosphere. It is transferred to a specially designed equipment that separates the biggest amount of the produced CO_2_. The separated CO_2_ is led to a storage tank, and the flue gas which has remained is vented to the atmosphere. Aside from flue gases, carbon capture from various industries, e.g., cement and stainless-steel manufacturing facilities, can also be classified as post-combustion capture, although these industries yield higher CO_2_ concentrations compared to the typical flue gases encountered in most post-combustion power plants. Nowadays, chemical absorption, which employs the utilization of aqueous amine solutions, is the most commonly applied post-combustion capture process [19,25].

### 2.2. Pre-Combustion Capture

Pre-combustion capture refers to CO_2_ capture from a synthesis gas (syngas) after the conversion of CO into CO_2_. This process initially involves the reaction of a fuel with O_2_ (or air) and/or steam to produce mainly a synthesis gas or a fuel gas, which is composed of CO and H_2_. CO then reacts with steam in a catalytic reactor, which is known as a ‘shift converter’, to form CO_2_ and more hydrogen in a process called water–gas shift reaction (WGS). After the WGS reaction, the CO_2_ content in the flue gas is high, i.e., in the range of 15–60% (dry basis), at a total pressure of 2–7 MPa, and physical solvents (e.g., Rectisol or Selexol) are usually employed for CO_2_ capture. The resulting fuel, which is rich in hydrogen, can be used for heat and power generation, e.g., in furnaces, boilers, engines, gas turbines and fuel cells. A 15–40% CO_2_ content at increased pressures (200–600 psi) is usually contained in most pre-combustion gas streams [19,20,25,26].

### 2.3. Oxy-Fuel Combustion Capture

Oxy-fuel combustion capture was initially developed to yield CO_2_ of high purity (>99%) during enhanced oil recovery (EOR). In this relatively new process, almost pure oxygen (95–99%), instead of air, is used for combustion, resulting in a flue gas that contains H_2_O and CO_2_ at a very high concentration. When pure oxygen is used to burn the fuel, the temperature of the flame is very high, but CO_2_ and the rich-in-water flue gas can be recycled in the combustor to change this. For the production of oxygen, cryogenic air separation is usually applied or, to a lesser extent, other alternative technologies (e.g., membrane separation) [19,25,26].

### 2.4. Capture from Industrial Process Streams

Several industrial applications involve process streams, which present the opportunity for capturing large quantities of CO_2_ at relatively low costs. CO_2_ capture from these sources has been applied for more than 80 years, although most of the captured CO_2_ is usually discharged into the atmosphere since there is no incentive for further utilization or storage. Capturing CO_2_ from industrial process streams may not be the complete answer to current climate change requirements since the amount of CO_2_, which is generated during combustion, is much higher; however, it can provide the ‘starting point’ for the initial CO_2_ capture. Current examples of CO_2_ capture from industrial process streams include the purification of natural gas and the production of syngas for the synthesis of ammonia, alcohols and synthetic liquid fuels. Other examples, which involve sources of CO_2_ that are usually not captured, include fermentation and cement and steel production facilities. Finally, there are industrial process streams which employ two or more of the aforementioned CO_2_ capture processes, regardless of whether they contain a low or high CO_2_ concentration [19,21].

## 3. Post-Combustion CO_2_ Capture Technologies

In comparison with the other CO_2_ capture processes (i.e., pre-combustion capture, oxy-fuel combustion capture and capture from industrial process streams), post-combustion capture is technologically more mature and presents the highest short-term potential for CO_2_ reduction, as it can be easily incorporated and adjusted to existing fossil-fueled power plants or implemented into other industrial CO_2_ emitters (e.g., cement industries, iron and steel production industries). This retrofit of existing power plants is related to the smaller interferences of the capture process with other components. However, the process should be appropriately adapted to treat impurities (mainly SO_x_ and NO_x_) and the considerable amounts of oxygen, which are contained in flue gases [26,27,28].

The main post-combustion CO_2_ capture technologies include chemical absorption (with liquid solvents), adsorption (with solid adsorbents), cryogenic separation and membrane separation [29,30]. Table 1 presents the main benefits and challenges of these technologies. Selecting the appropriate capture technology depends on the specific discharge conditions, and the main criterion is the flue gas state, i.e., its composition, flow rate, temperature and CO_2_ content. This selection is also affected by the desired production (e.g., CO_2_ purity and transport pressure) and the discharged standards (e.g., H_2_S, NO_x_ and SO_x_) [31]. The CO_2_ capture technologies which have been adequately developed and applied commercially (TRL9) include chemical absorption and cryogenic separation. Chemical absorption allows CO_2_ capturing from streams with a low CO_2_ content and achieves high CO_2_ capture efficiencies. Cryogenic separation is suggested for increased CO_2_ concentrations (>90%) and produces high-purity liquified CO_2_. However, these technologies are highly energy-demanding. On the contrary, membrane technologies, which employ the pressure difference as a driving force to achieve CO_2_ separation, may become a more efficient option, especially when the treatment of CO_2_ stream (with a concentration of >10%) is coupled with the appropriate tuning of the driving force. In addition, separation with membranes does not require storing or treating of hazardous chemicals, although it may present a lower separation efficiency than chemical absorption and cryogenic separation. Currently, post-combustion CO_2_ capture from flue gases with membrane separation is on a pilot-scale level (TRL-6) [32].

It should be noted that chemical absorption and membrane separation cannot be directly compared in terms of energy consumption due to the different energy sources (i.e., heat for absorption and electricity for membrane). Similarly, an overall cost comparison of CO_2_ capture technologies would also be difficult, and each technology should be examined case by case, taking into account the specific conditions that are applied, such as flue gas composition, flue gas flow rate and targeted CO_2_ capture capacity. Nonetheless, comparative (techno-)economic assessments have been conducted for the main CO_2_ capture technologies. According to Hongjun et al. [33], who compared, in 2011, the cost of chemical absorption, membrane separation and Pressure Swing Adsorption (PSA) for CO_2_ capture from flue gases of coal-fired power plants, the cost ranged between USD 30 and USD 60 per ton for chemical absorption, between USD 50 and USD 78 per ton for membrane separation and between USD 40 and USD 63 per ton for PSA. In 2017, after reviewing chemical absorption and membrane separation for their energetic and economic performances, Wang et al. [30] concluded that (i) chemical absorption still remains energy-intensive and costly, despite the diversity of optimization methods that are applied to reduce the energy consumption and cost of this technology; (ii) although membrane separation was initially expected to compete with chemical absorption, it presents advantages only at a low CO_2_ capture percentage (<90%), and its capture ability is limited by the characteristics of the employed membrane materials; and (iii) it is difficult to compare the energetic and economic results in the literature and infer general conclusions since the relevant research studies are usually based on specific conditions and assessments. Four years later (2021), Zanco et al. [8] compared chemical absorption, adsorption (with zeolite) and membrane separation for post-combustion CO_2_ capture from flue gases. The comparative assessment showed that adsorption and membrane separation can become cost-competitive on a small scale (i.e., <100 tons of processed flue gas per day) with low recovery rates (i.e., <40%); however, chemical absorption remains the most cost-effective option for the majority of facilities and recovery rates. In 2022, the cost for carbon capture projects was estimated globally at USD 60–110/per ton, and it is expected to decrease further to USD 30–50 per ton by 2030 [34].

### 3.1. Chemical Absorption

Absorption is the most applied technology for CO_2_ capture because it is technologically mature, commercially available and easily adaptable to several processes, such as post-combustion, pre-combustion and oxy-fuel combustion capture. In this technology, CO_2_ is selectively absorbed from a flue gas by means of a lean solvent via a physical or chemical mechanism. Physical absorption is based on the solubility of carbon dioxide in the solvent, and chemical absorption is based on the chemical reaction between carbon dioxide and the solvent. The latter is usually preferable for CO_2_ capture from power plants as it presents higher CO_2_ selectivity at low CO_2_ partial pressures [35,36].

**Figure 2:**
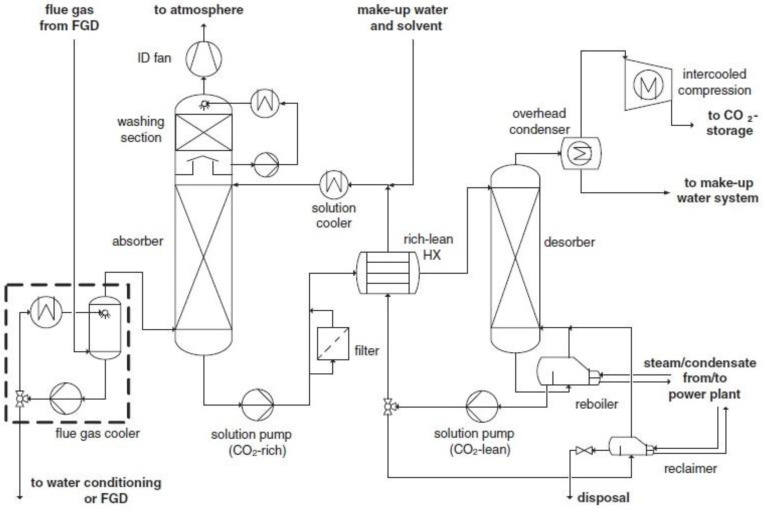
Simplified flow chart of chemical absorption technology for CO_2_ capture [28].

In chemical absorption technology (Figure 2), first, CO_2_ is chemically absorbed into a lean solvent in an absorption column (absorber), and then it is desorbed by utilizing a stripping gas of high temperature in a column where the solvent is regenerated (desorber or stripper), consuming a significant amount of energy. More specifically, the cooled flue gas initially enters the absorber and contacts, in a counter-current flow, the descending solvent, which is usually a 15–40 wt % MEA aqueous solution. The solvent becomes ‘rich’, i.e., absorbs CO_2_, at 40–60 °C and 1 bar, and exits the absorber, while the clean flue gas exists from the top of the column before it is passed to a wash column and vented to the atmosphere. The rich solvent is then heated in a cross-flow heat exchanger (through the regenerated later-coming hot lean solvent from the desorber) and pumped to the top of the desorber where it is regenerated at increased temperatures (100–120 °C) and at a pressure of 1.5–2 atm. Heat is provided by a reboiler, which is the most important energy penalty of the process. The regenerated solvent is finally pumped back to the absorber via the cross-flow heat exchanger, which decreases the temperature [35,37].

Various solvents are used in the absorption technology, namely amines, ionic liquids, ammonia, deep eutectic solvents and water-lean solvents. Monoethanolamine (MEA) is one of the most employed (and studied) solvents on the lab, pilot and full scales. MEA presents low cost, viscosity and volatility, which are considered desirable properties for CO_2_ capture. In addition, a significant amount of data/information is available regarding its physical and chemical properties (degradation, solubility of O_2_, etc.), both in lab-scale reactors and in pilot-scale or industrial capture plants. The most important drawback, however, is the high energy amounts that are needed to break the bonds of the formed carbamate (CO_2_ + MEA) and regenerate the solvent. This is almost 57.5% of the total energy consumption, accounting for 50% of the operating cost [38,39,40]. Furthermore, the incorporation of such systems in a power plant increases almost 70–80% of the electricity cost and reduces 25–30% of the net efficiency of the power plant [39,41]. MEA is also highly oxidative and easily degrades when it comes in contact with impurities, such as excess oxygen and sulphur dioxide. This degradation accounts for almost 10% increase in the operating cost. Consequently, the relevant research studies aim to either decrease the high consumption of energy via the application of alternative solvents and process configurations, or to employ less energy-intensive CO_2_ capture technologies where solvent regeneration is not required (e.g., membrane separation) [35,42].

### 3.2. Adsorption

**Figure 3:**
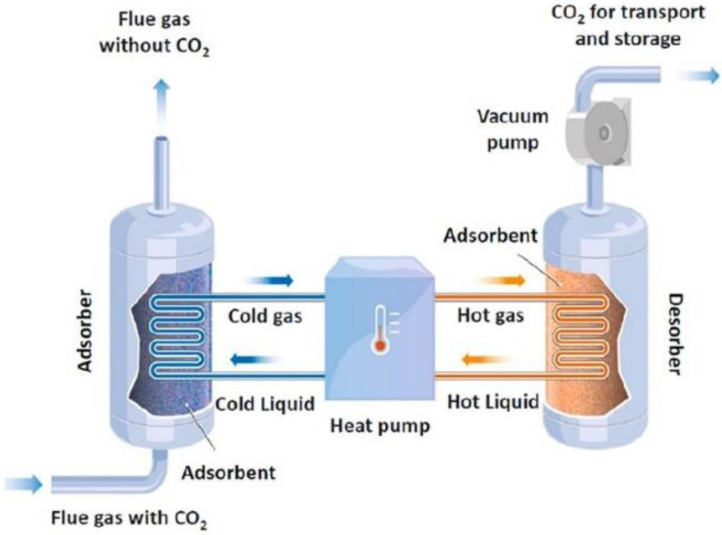
Schematic of adsorption technology for CO_2_ capture [48].

In this technology, an adsorbent agent is used to selectively adsorb and separate CO_2_ from the flue gas in a two-step process (Figure 3); CO_2_ is initially adsorbed on the surface of the adsorbent agent, which is then regenerated usually via the application of heat (Temperature Swing Adsorption, TSA) or by reducing the pressure (Pressure Swing Adsorption, PSA) [43,44]. For TSA, the energy consumption derives from heating, while for PSA, the energy consumption mainly comes from the compressor system [45]. It is reported that TSA processes offer additional advantages compared to PSA. First of all, the flue gas, which is emitted at an almost ambient pressure, does not require pressurization. Secondly, waste heat can be used to provide the energy that is needed for temperature swing. Therefore, the low temperature difference between the regeneration and adsorption steps of TSA (30–150 °C) and the possibility to use the available waste heat reduce the operating cost, thereby promoting the implementation of TSA [46]. Nowadays, TSA is mainly employed for post-combustion capture of CO_2_ from flue gases [47].

Adsorption can be characterized as physical or chemical, depending on the involved mechanism. During physical adsorption (or *physisorption*), CO_2_ molecules attach to the pore walls of the adsorbent agent primarily through microscopic forces (Coulomb force and Van der Waals force), without forming chemical bonds. At ambient temperature, CO_2_ physisorption takes place and the gas uptake is directly related to the porous structure of the adsorbent’s surface. Chemical adsorption (or *chemisorption*) refers to the formation of chemical bonds between CO_2_ and the adsorbent surface. During chemisorption, coating or chemical grafting occurs on the surface of a porous material by integrating basic groups that interact with the acidic CO_2_ molecules. The adsorption of CO_2_ at increased temperatures (>140 °C) is primarily governed by chemisorption. In the temperature range of 25–140 °C, both physisorption and chemisorption can occur [44,49].

The selection of the adsorbent agent is one the most important factors to achieve effective separation when the adsorption technology is considered for CO_2_ capture. The adsorbent agent should have high CO_2_/N_2_ selectivity, high adsorption capacity, fast kinetics, high surface area, mild desorption ability, high resistance/tolerance towards moisture and impurities, high mechanical strength, low operating cost and high stability when applied in a multi-cycle operation [50]. Typical adsorbents for CO_2_ capture are zeolites, activated carbon, alumina, silicates and metal–organic frameworks (MOFs) [51,52].

The application of adsorption can result in high energy savings compared to the commonly applied absorption with amines. In addition, it is a simple, environmentally friendly technology that is quite readily retrofitted to existing plants and, thus, offers the flexibility to capture CO_2_ from different industrial CO_2_ sources due to the different available adsorbent regeneration modes and reactor types [51,53]. Other advantages include high adsorption capacity at ambient conditions, stability for long-term application, low cost for regeneration and fast kinetics [50]. However, although adsorption is a relatively mature technology for some industrial applications of large scale, its application in real-field post-combustion CO_2_ capture process still presents significant challenges concerning the adsorption materials, the gas–solid contact systems and the regeneration mode. In addition, the flue gas used should be rich in CO_2_ because the majority of the available adsorbents have low selectivity. For this reason, current research studies have focused mainly on the development of innovative adsorbent materials and aimed to reduce energy consumption by minimizing the adsorption heat and maximizing the adsorbents’ capacity [37,53].

### 3.3. Cryogenic Separation

**Figure 4:**
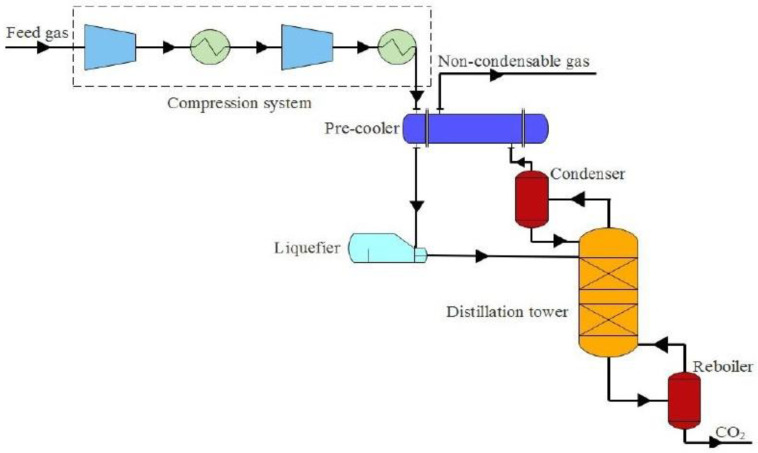
Process flow diagram of cryogenic separation technology for CO_2_ capture [57].

Cryogenic separation (Figure 4) exploits the condensation (cryogenic distillation techniques) or desublimation points (cryogenic desublimation techniques) of the gases that are contained in a flue gas. In the first group of techniques, distillation columns are employed to separate and recover CO_2_ in liquid form. The obtained CO_2_ is of high purity; however, these techniques are highly energy-intensive because very high pressures are required to prevent the formation of CO_2_ frost. During desublimation techniques, CO_2_, which is at atmospheric pressure and a temperature of −78.5 °C, desublimates directly from the gaseous state to the solid state [54].

When compared to the other CO_2_ capture technologies, the principal advantage of cryogenic separation is the high recovery rate and purity. In addition, it is a relatively simple technology that employs a two- or three-step sequence (compression, expansion and separation); it does not demand the utilization of volatile chemicals (e.g., as in absorption); and it is conducted under mild pressures [29,55]. However, cryogenic separation demands high power to operate the refrigeration unit, which increases the operating cost, and it also requires feed gas pre-treatment and dehydration to avoid CO_2_ freezing in the cold section of the fractionation equipment [56].

### 3.4. Membrane Separation

During membrane separation, CO_2_-selective membranes are utilized to separate CO_2_ from a flue gas stream. CO_2_ capture with membrane separation was applied initially for the purification of natural gases, as an alternative to the utilization of amine solvents, in the 1980s. Since then, membrane separation technologies have expanded their market share due to a series of benefits, such as low capital cost, low energy consumption, low space requirements and high sustainability in distant areas [10,58]. In addition, they are environmentally friendly, have simple operation and do not produce harmful wastes, as opposed to, e.g., chemical absorption which employs amine absorbents [59]. The following section presents the basic principles, the main process configurations and membrane materials, and the current advancements regarding the application of membrane-based technologies, mainly for efficient CO_2_ capture from exhaust flue gases.

## 4. CO_2_ capture with Membrane Technologies

### 4.1. Basic Principles and Mechanism of Membrane Gas Separation

**Figure 5:**
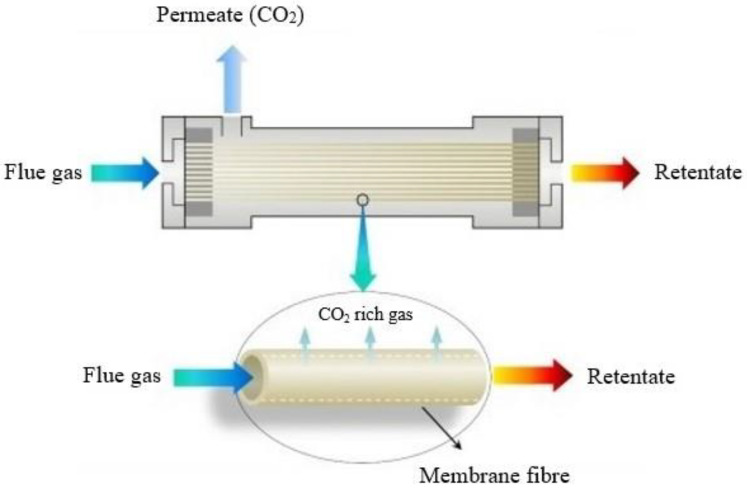
CO_2_ separation principles with the use of a membrane module.

During gas separation using membrane technology, a membrane acts as a filter that allows specific molecules to permeate (e.g., CO_2_) but prevents other molecules from entering the membrane (e.g., CH_4_ and H_2_O) (Figure 5) due to characteristics such as gas–membrane chemical interactions or the kinetic diameter. Membrane processes, such as micro-filtration, ultra-filtration, nano-filtration and reverse osmosis, are widely used in solid–liquid separations [60]; however, membrane gas separation is also attracting intensive research for carbon capture, utilization and storage (CCUS) during recent years [10,61].

For membranes with no permanent porosity, i.e., membranes that consist of dense polymeric materials, the most widely adopted mechanism/model for mass transport is the solution-diffusion model. According to this model, transport occurs in three steps: (1) dissolution or sorption of a gas into the membrane at the high-pressure material side, (2) diffusion of the sorbed gas through the membrane, and (3) desorption of the gas from the membrane at the low-pressure material side. The chemical potential difference between the high-pressure and low-pressure contacting phases controls the driving force, which is created through gas compression or vacuum. For ideal gas contacting phases, the gas flux across the membrane is calculated using the following equation:

(1) $$J_{i}=\frac{Q_{i}}{\delta }\cdot (P_{r}\cdot x_{i}-P_{p}\cdot y_{i})$$

where *J_i_* is the flux across the membrane of species *i* (mol/m^2^/s); *Q_i_* is the membrane permeability for species *i* (mol·m/m^2^/s/Pa); *δ* is the effective membrane thickness (m); *P_r_* and *P_p_* are the feed/retentate high pressure and the permeate low pressure (Pa), respectively; and *x_i_* and *y_i_* are the high-pressure and low-pressure gas-phase mole fractions (mol/mol), respectively. The permeability is equal to the product of gas solubility and diffusivity in the membrane. The ratio of permeability to membrane thickness in Equation (1) is defined as the gas permeance: *q* ≡ $\frac{Q_{i}}{\delta }$ · (mol/m^2^/s/Pa). Commonly, permeance is expressed in gas permeation units (GPUs), where 1 GPU = 3.35 · 10^−10^ (mol/m^2^/s/Pa).

**Figure 6:**
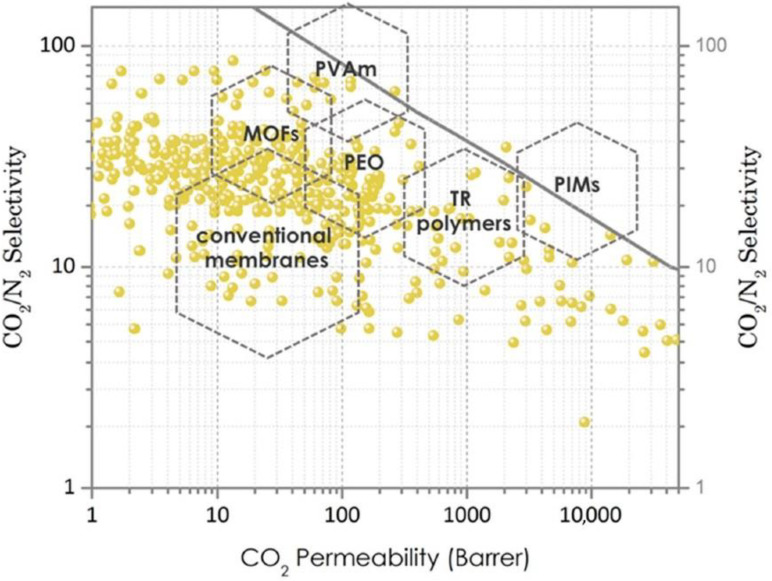
Robeson upper bound plot for gas separation membranes made from various materials (PVAm: polyvinylamine, MOFs: metal–organic frameworks, PEO: polyethylene oxide, TR: thermally rearranged polymers, PIMs: polymers of intrinsic microporosity) [63].

Selectivity expresses the ability of a membrane to separate two gases. For a pair of gases, *i* and *j*, selectivity is defined as the ratio of gas permeabilities or permeances:

(2) $$a_{i}{,}_{j}=\frac{Q_{i}}{Q_{j}}=\frac{q_{i}}{q_{j}}$$

where component *i* is the gas with the higher permeability, resulting in a selectivity greater than 1. In membrane technologies, selectivity and permeability are the most important parameters for efficient gas separation and determine the process economics; the energy (operating) cost is controlled by selectivity, whereas the membrane area (capital) cost is controlled by permeability. An increase in selectivity decreases the amount of gas that must permeate from the high-pressure feed to the low-pressure permeate to achieve the desired targets for product purity; this decreases the compression energy lost due to permeation. An increase in permeability or permeance, i.e., of the gas permeation flux per unit of driving force, reduces the area of the membrane and the capital cost that is necessary to achieve a specific feed or product flow rate [14,62]. The Robeson correlation is an empirical correlation that presents a trade-off between permeability and selectivity of gases, and its upper boundary is usually employed to assess the performance of membrane systems (Figure 6). To address the challenges in reducing carbon capture cost, a membrane material should be on the Robeson upper bound (or above it), i.e., in the region of high permeability/moderate selectivity [63,64].

### 4.2. Membrane Configurations and Process Engineering

**Figure 7:**
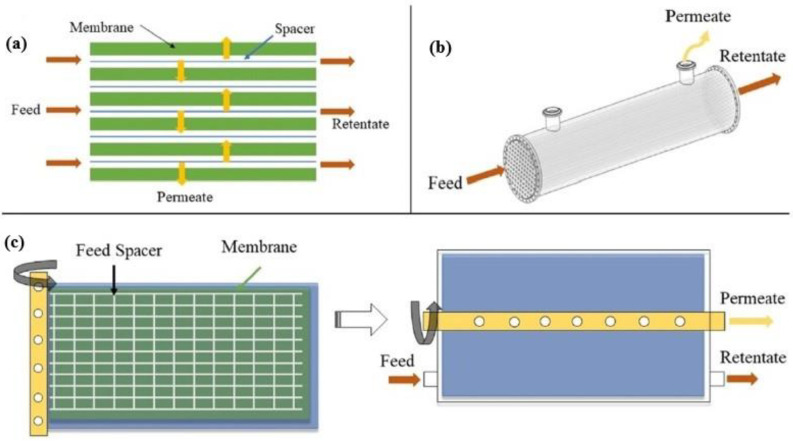
Graphic illustration of the main membrane configurations: (**a**) flat-sheet membrane in cross flow, (**b**) hollow fiber membrane in co-current flow, and (**c**) spiral-wound membrane before and after spinning [65].

Typical membrane configurations for separating CO_2_ include hollow fiber (HF), flat-sheet (FS) and spiral-wound (SW) membranes (Figure 7). Hollow fiber membranes, which contain hundreds or thousands of hollow fibers packed into bundles, are the most studied membrane configuration due to their high surface area per unit volume, which promotes gas transfer. However, they present drawbacks, such as fiber fouling and significant pressure drops. On the contrary, flat-sheet membranes, which are usually stacked on top of each other, present lower pressure drops and enhanced mass transfer because they employ specially designed feed spacers. In addition, they can be easily fabricated and physically or chemically cleaned. Spiral-wound membranes actually consist of flat-sheet membranes that are rolled around a collection tube [65,66].

**Figure 8:**
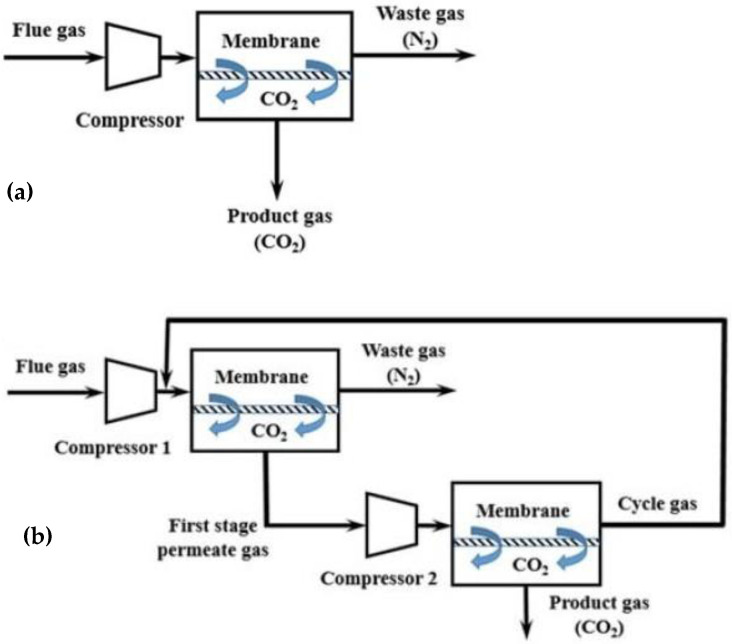
Process engineering configurations for CO_2_ capture from flue gases using membranes: (**a**) single-stage membrane configuration, and (**b**) two-stage membrane configuration [69].

According to the employed process engineering configuration, gas separation using membranes can occur in a single-stage process with one membrane module or in a two- (or multi-)stage process with two (or more) membrane modules that are placed in series or in a parallel configuration (Figure 8) [67,68]. During the single-stage process (Figure 8a), high selectivity is needed to achieve 95% purity and 90% recovery of CO_2_. Consequently, it is difficult to achieve high targets using the single-stage membrane process, mainly because the purity of the final product is limited by the low CO_2_ content in the feed gas and by the trade-off effect between recovery and purity. On the contrary, high purity and recovery targets are achieved more easily in the two-stage membrane process (Figure 8b) as the gas recycling enhances the recovery of CO_2_ significantly. However, the two-stage membrane process consumes more power since more compressors (or vacuum pumps) are needed, and the gas recycling increases the required membrane area. Consequently, during membrane separation with two or more stages, the main target is to reduce energy consumption and membrane area [69].

### 4.3. Membrane Materials

Each membrane material presents advantages and disadvantages, which are concerned with the separation performance, material cost, lifetime and other characteristics, such as chemical and thermal stability, and mechanical strength. Organic (polymeric) membranes, inorganic membranes and mixed-matrix membranes (MMMs) are the main membranes applied for post-combustion CO_2_ capture [70,71,72].

Among various membrane materials, polymeric materials present inherent advantages in terms of cost, variety and ease of processing. Polymers, including polyacetylene [73], polyaniline [74], polyamides [75], polyimides [76], polyetherimides [77], polycarbonates [78], poly(phenylene oxides) [79], poly(ethylene oxides) [80], polysulfones [81] and cellulose acetate [82], have been examined for post-combustion CO_2_ capture. Generally, the solution-diffusion transport mechanism and the facilitated transport mechanism are broadly adopted as the principal mechanisms when the design of new polymers is considered. Membranes which are made from the aforementioned polymeric materials follow the solution-diffusion mechanism. In facilitated transport membranes, CO_2_ transport is enhanced by the interaction between CO_2_ molecules through reversible reactions (see Section 5.5). Compared to other materials, polymeric materials can be regarded as the optimal materials due to many characteristics, such as thermal stability, mechanical strength and chemical resistance. By controlling the polymer preparation and chemical composition process, the permeability and selectivity of these membranes can be easily adjusted. In addition, polymeric membranes are one of the best options due to the development of membrane technologies in various industries, such as biogas upgrading and petrochemicals. However, CO_2_ adsorption via polymer-based materials can cause swelling and plasticization problems [15,83]. Table 2 presents the CO_2_/N_2_ separation performance for various polymer-based membranes.

Non-polymeric materials, such as activated carbon, zeolites, silica and metal–organic frameworks (MOFs), are also emerging for CO_2_ capture. These membranes are more stable than polymeric membranes and, therefore, they are strong candidates for the efficient separation of gas mixtures, especially under harsh operating conditions. However, although inorganic membranes can be used in adverse conditions, the construction and sealing of the relevant modules for applications of high temperatures are quite difficult, and the cost of production is often much higher in comparison with polymer membranes. Ceramic membranes, which usually consist of aluminum oxide (Al_2_O_3_), titanium oxide (TiO_2_) or carbon nanotubes (CN), are a group of inorganic membranes with improved properties in terms of mechanical strength, thermal stability and chemical stability. Nonetheless, they present short operating time and low flexibility to form HF or SW membranes; as a result, their utilization in CO_2_ capture is still under research [72,83,92].

The limitations of membranes that are made from pure polymeric or inorganic materials prevent their widespread utilization in gas separation processes. Although pure polymeric membranes present exceptional mechanical properties, which allow their easy processing, they are limited by the trade-off effect that prevents achieving both high selectivity and permeability. Pure inorganic membranes present high selectivity or permeability, but they are thick and fragile and, thus, it is difficult to use them on a full-scale level. Aiming to overcome the aforementioned issues, the co-blending of organic and inorganic materials has been proposed to fabricate composite or mixed-matrix membranes (MMMs) with high permeability, selectivity, enhanced mechanical properties and potential for large-scale implementation. As a result, current research in membrane-based gas separation focuses mainly on the synthesis of composite membranes, which are usually fabricated via the integration of innovative inorganic materials, also known as ‘fillers’, into polymeric membranes [93,94]. The following section presents the latest developments regarding the use of these membranes for separating CO_2_.

## 5. State of the Art in CO_2_ Capture with the Use of Membrane Technologies

The application of membrane separation processes for CO_2_ capture is increasingly gaining traction during the last decade. Membrane technologies are environmentally friendly, energy-efficient and easily scalable, while also presenting cost-effectiveness and design simplicity [95]. As concerns carbon capture, membrane technologies are employed mainly for H_2_/CO_2_ separation during pre-combustion, CO_2_/N_2_ separation during post-combustion and O_2_/N_2_ separation during oxy-fuel combustion. This section focuses primarily on the latest developments of membrane separation processes for CO_2_/N_2_ separation during post-combustion capture.

The most recent advances in CO_2_ capture using membrane technologies principally involve employing a membrane material; the vast majority of research studies that examine membrane-based technologies for post-combustion CO_2_ capture focus on the fabrication and application of novel membrane materials which selectively separate CO_2_ from N_2_. In most of them, a polymeric material (continuous phase) is combined with an inorganic material of micro- or nano-size (filler, dispersed phase) to form ***composite membranes or mixed-matrix membranes (MMMs)*** with improved properties, i.e., enhanced intrinsic and surface characteristics. To achieve this, composite membranes employ a broad variety of materials, such as silica, zeolites and metal oxides [58,83,96,97].

The fillers, which are incorporated in MMMs, can be classified as non-porous (e.g., metal oxides and silica) or porous [98]. Generally, porous materials are classified into four groups [99,100]:

(i) Inorganic materials, e.g., zeolites.
(ii) Carbon-based materials, e.g., carbon nanotubes and carbon molecular sieves (CMSs).
(iii) Organic-based materials, such as (a) porous organic frameworks (POFs), which include covalent organic frameworks (COFs), porous aromatic frameworks (PAFs), covalent organic polymers (COPs) and porous organic polymers (POPs), and (b) microporous polymers, which include polymers of intrinsic microporosity (PIM) and thermally rearranged (TR) polymers.
(iv) Hybrid materials, which are also known as metal–organic frameworks (MOFs).

Among the aforementioned materials, recent progress focuses mainly on the development and integration of metal–organic frameworks (MOFs) and carbon molecular sieves (CMSs) into novel MMMs. Other types of MMMs, which are increasingly examined for their potential to improve CO_2_ separation, include nanocomposite membranes, which employ nano-sized fillers (of various materials); ionic liquid (IL)-based membranes, which employ ionic liquids as the continuous phase in the fabricated MMMs; and facilitated transport membranes (FTMs), where the gas diffusion mechanism is based on facilitated transport.

### 5.1. Metal–Organic Framework (MOF) Membraness

Metal–organic frameworks (MOFs) comprise a new kind of porous materials, which are constructed from multidentate organic ligands and metal ions. In comparison with typical porous materials, e.g., zeolites and carbon nanotubes, MOFs can provide an ideal platform for gas absorption and separation, drug delivery, catalysts, electrode materials and semi-conductors due to their well-ordered architecture. MOFs present characteristics such as tunable pore size, large surface area and chemistry, which are favorable for gas separation and for unlocking efficient CO_2_ capture paths [101].

**Figure 9:**
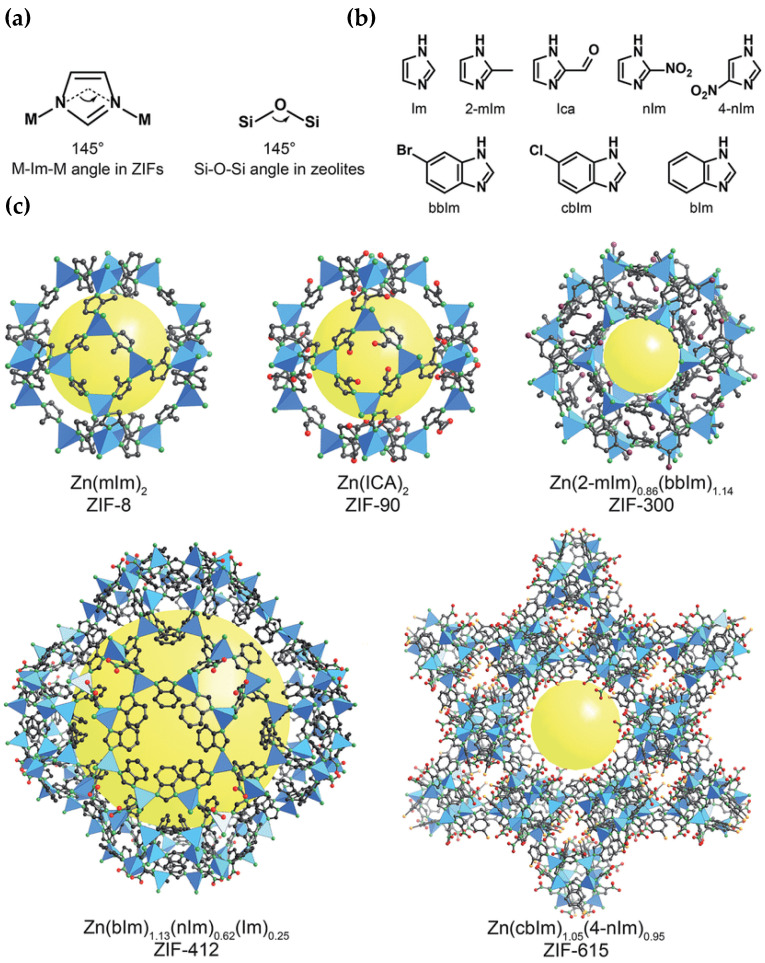
Zeolitic imidazolate frameworks (ZIFs): (**a**) design of a ZIF using tetrahedral metals and imidazolates to present tetrahedral topologies typically found in zeolites, (**b**) main imidazolate linkers which are applied during ZIF synthesis, and (**c**) crystal structures of a ZIF with various pore sizes and openings. Atom labelling scheme: C, black; O, red; N, green; Br, purple; Cl, orange; and Zn, blue polyhedra. H atoms are not depicted for clarity. Yellow spheres represent the space in the framework [107].

Apart from the development of typical fabrication techniques for MOF-based membranes, membranes that combine MOFs with other materials have also been generated in recent years. MOF membranes, which usually have a thickness of micrometers, present high mechanical stability; however, they have low separation performance due to high mass transfer resistances and result in low permeabilities. To deal with this drawback, ultrathin two-dimensional monolayer MOF membranes with a thickness of nanometers have been proposed [102,103]. Yao et al. (2023) [104] employed an anodic electrodeposition method to incorporate in situ different kinds of MOFs (HKUST-1, Cu-BDC and Cu-BDC-NH_2_) into the nanochannels of graphene oxide (GO), aiming to result in the formation of a new layer-by-layer structure confined by GO layers. The pore-size distribution of the obtained membranes was wider, and a significant increase in elastic modulus and hardness was observed. The membranes also presented high CO_2_ capture capacity and selectivity, providing a promising strategy to produce functional and mechanically strong MOFs for real-field applications. One of the most representative MOFs, which presents high affinity for CO_2_ due to its abundant CO_2_-philic groups and sites, is zeolitic imidazolate framework-8 (ZIF-8) (Figure 9). ZIF-8 was incorporated as a filler by Wang et al. (2024) [105] into a Pebax matrix (60 wt % polyethylene oxide and 40 wt % polyamide 6), and the obtained MMM achieved enhanced CO_2_ separation performance. Other novel MOF-based membranes, which offer significant benefits, e.g., enhanced separation performance, defect-free structures and improved mechanical properties, include ionic liquid (IL)-MOF membranes, covalent organic framework (COF)-MOF membranes and MOF-glass membranes [106].

### 5.2. Carbon Molecular Sieve (CMS) Membranes

Carbon molecular sieves (CMSs) are a type of activated carbons with pores of a very low (molecule) size, which have been utilized for separating O_2_ from air and CO_2_ during biogas upgrading. However, they are much less investigated for CO_2_/N_2_ separation and carbon capture from flue gases [108]. The rigid pore structure of CMS membranes presents a bi-modal pore-size distribution, where the micropores (7–20 Å) improve gas permeability and the ultra-micropores (<7 Å) enhance gas selectivity through molecular sieving. Due to these properties, CMS membranes are often utilized for separating gases with similar molecular kinetic diameters [109,110]. CMS membranes have attracted worldwide attention as they exhibit higher chemical and thermal stability and enhanced gas separation performance compared to polymeric membranes. Several polymers are used to synthesize CMS membranes, such as polyimides, phenol formaldehyde, cellulose, poly(phenylene oxide), sol–gel polymers, poly(vinylidene chloride-co-vinyl chloride) and poly(furfuryl alcohol). Among the aforementioned materials, poly(furfuryl alcohol) is considered a strong possible precursor for manufacturing high-performance CMS membranes [111,112].

**Figure 10:**
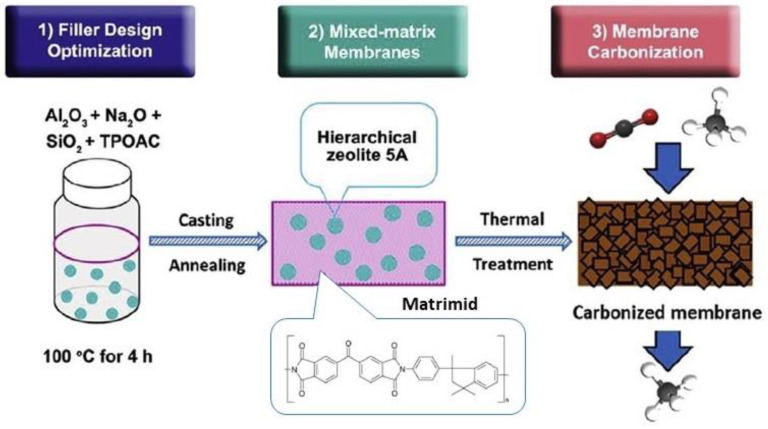
Synthesis procedure of CMS membranes [114].

The development of MMMs which contain CMSs has been examined by various researchers. A suitable filler must withstand high temperatures and be easy to prepare, taking into account the preparation conditions of CMS membranes and the demand for economic efficiency [113]. Li et al. (2019) [114] adopted the strategy of filler design optimization with membrane carbonization to produce mixed-matrix CMS membranes incorporating a hierarchical zeolite 5A filler (Figure 10). Apart from its intrinsic micropores, the hierarchical filler also possessed mesopores of ∼8 nm that offered additional transport pathways to facilitate the diffusion of CO_2_ through the membrane carbon matrix. More recently, Zhang et al. (2023) [113] incorporated the zeolite ZSM-5 into the carbon matrix of a CMS membrane to fabricate a CMS/ZSM-5 MMM. The results showed that the gas permeabilities of CMS/ZSM-5 MMM for H_2_, CO_2_, O_2_, N_2_ and CH_4_ were significantly improved in comparison with the pure CMS membranes.

### 5.3. Nanocomposite Membranes

When the incorporated inorganic material (filler) is of nano-scale size, the obtained MMMs are also known as nanocomposite membranes. Nanofillers (usually between 1 and 100 nm) are increasingly incorporated in polymeric matrices to produce MMMs in recent years. Nanofillers present molecular sieving ability, an arranged pore structure, good mechanical properties and thermal stability, and their exceptional interfacial compatibility forms special nano-channels for the transportation of CO_2_ when they are combined with polymers. Nanofillers in MMMs are used to decrease the gas transport resistance and enlarge the chain spacing in the polymer. In addition, they increase CO_2_ solubility in MMMs, while the sieving ability of their porous structure improves significantly the gas selectivity and permeability [95].

**Figure 11:**
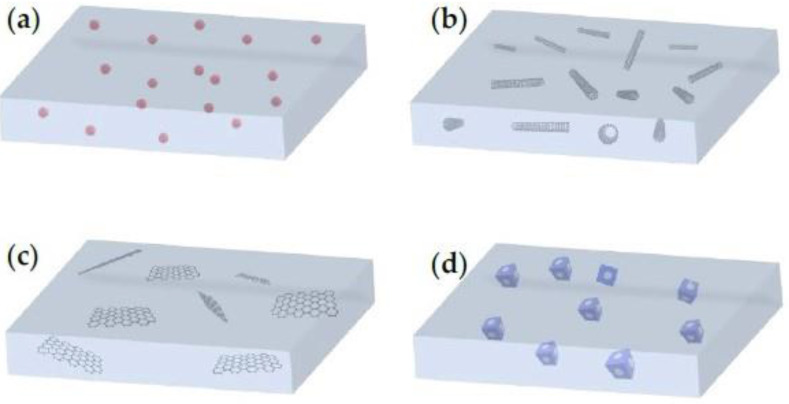
Mixed-matrix membranes (MMMs) with (**a**) 0D nanomaterials (nanoparticles), (**b**) 1D nanomaterials (e.g., carbon nanotubes), (**c**) 2D nanomaterials (e.g., graphene oxide nanosheets) and (**d**) 3D nanomaterials (e.g., microporous nanomaterials) [115].

Based on the number of dimensions, nanofillers (and their respective MMMs) can be zero-dimensional (0D), one-dimensional (1D), two-dimensional (2D) and three-dimensional (3D) (Figure 11). Zero-dimensional nanofillers are typically represented by nanoparticles, which include mainly gold, zinc, silver or metal oxides with a pore size of 1–50 nm usually. One-dimensional nanotubes, nano-wires, nano-rods and nano-fibers are nanomaterials of ‘needle’ shape, while two-dimensional nanomaterials are thin nanosheets with only one external nano-scale dimension. Two-dimensional nanomaterials can reach a few square microns, usually far exceeding their thicknesses. Nanoporous materials, such as zeolites, silicalites and MOFs of polycrystalline structures can be regarded as 3D nanofillers. It must be mentioned that bulk nanoparticles and bundles of 1D materials and multi-nanolayers are also regarded as 3D nanomaterials and, therefore, exhibit tunable properties based on their dispersion state [115].

In recent years, various research studies have investigated the fabrication and utilization of nanocomposite membranes for efficient CO_2_ capture. Dai et al. (2023) [116] demonstrated that the addition of one-dimensional carboxymethylcellulose (CMC) between 2D g-C_3_N_4_ nanosheets is a promising material for CO_2_ separation membranes. Using the electrostatic self-assembly method, Zhao et al. (2023) [101] synthesized novel porous amino-functionalized nanosheets with polyethyleneimine (PEI-F-Ce) and combined them with a polyethylene oxide (PEO) matrix to fabricate an MMM (XLPEO/PEI-F-Ce) of high CO_2_/N_2_ selectivity. It was shown that XLPEO/PEI-F-Ce selectivity was significantly enhanced due to the combined improvement in solubility and reaction selectivity. Maleh et al. (2022) [117] suggested a novel polyethersulfone (PES)-based MMM for separating CO_2_ from natural gases and flue gases. These researchers also studied how polyurethane (PU) and clay nano-sheets affect the structure and the separating properties of three MMMs that were based on PES, and showed that the incorporation of PU and clay nano-sheets into the PES matrix significantly improved its gas permeation and separation properties.

### 5.4. Ionic Liquid (IL)-Based Membranes

Ionic liquids (ILs) are molten organic salts in liquid form, with typical melting points lower than 100 °C [118]. ILs are attracting attention in solvent-based post-combustion CO_2_ capture because they present some remarkable characteristics, such as high thermal stability, flammability and low volatility with huge tunability when selecting cations and anions. Due to these properties, high CO_2_ solubility and high separation potential for specific gas molecules can be achieved. However, pure ILs present a small surface area and a limited capacity for CO_2_ capture [119]. Recently, the combination of ILs with membranes has been reported as a potential separation system. With membrane support, an IL can keep its solvent properties and, consequently, improve its gas separation efficiency. Several distinct types of membrane processes combined with ILs have been investigated, including supported ionic liquid membranes (SILMs), IL composite polymer membranes (ILPMs), IL composite mixed-matrix membranes (ILMMM), poly(ionic liquid) membranes (PILMs), IL gel membranes (ILGMs) and IL membrane contactors (ILMCs). These systems have shown high gas separation efficiency [120].

**Figure 12:**
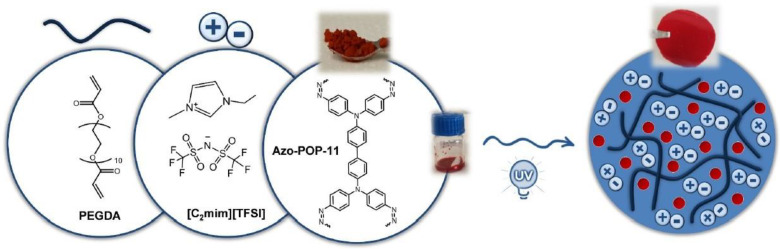
MMM containing azo-POP in ion gel [121].

In recent years, the number of research studies which employ ILs for the fabrication of MMMs with enhanced CO_2_ separation characteristics has increased. Mahboubi et al. (2023) [118] combined 1-butyl-3-methylimidazolium acetate, as an ionic liquid, with a polyether-block-amide polymer and aluminium oxide (Al_2_O_3_) nanoparticles, and prepared a novel ternary MMM with improved permeability and selectivity for CO_2_. Nabais et al. (2022) [121] also suggested the incorporation of different azo-porous organic polymers (azo-POPs) (Figure 12), as fillers, into ion gels with a high IL content (80 wt %) for the fabrication of MMMs with enhanced CO_2_ separation characteristics. Ahmad et al. (2018, 2021) [122,123] successfully employed an IL as the continuous phase in an MMM and enhanced its CO_2_ separation performance. According to Sanni et al. (2022) [120], among the membrane–ionic liquid systems, a hybrid system that is comprised of cellulose acetate, methyl ammonium nitrate and graphene oxide (CA-methyl ammonium nitrate-GO) is a possible candidate with high CO_2_ capture (>80%) due to its enhanced porosity, mechanical and thermal stability, good selectivity and low CO_2_ permeability.

### 5.5. Facilitated Transport Membranes (FTMs)

**Figure 13:**
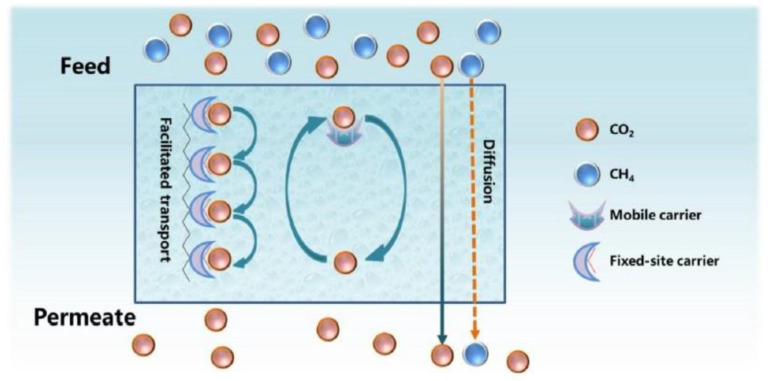
Gas transport through a facilitated transport membrane [124].

When CO_2_ permeates through a membrane, different transport mechanisms can take place, such as solution diffusion, Knudsen diffusion, convective diffusion, capillary condensation, molecular sieving and facilitated transport. In polymeric membranes, apart from the solution-diffusion mechanism, which is a common gas diffusion mechanism, especially in non-porous polymers, CO_2_ transport can be facilitated via reversible chemical reactions between CO_2_ and specific molecules (carriers) (Figure 13) [124]. These membranes, which are known as facilitated transport membranes (FTMs), can reversibly interact with CO_2_ and, thus, provide an ‘extra’ mechanism to promote its transport, while other components exclusively pass through the solution-diffusion model [60,94]. FTMs have shown great potential for superior gas separation performance and specifically for CO_2_ capture [125]. Through the incorporation of carrier agents into the polymer matrices to react with CO_2_ reversibly, FTMs provide high flux and selectivity. *Mobile carriers* and *fixed carriers* are the two predominant FTM types. A mobile carrier is also called a supported liquid membrane (SLM) or an ‘immobilized’ liquid membrane (ILM). This carrier initially reacts with CO_2_ on the feed side, and the subsequent product moves across the membrane. On the permeate side, CO_2_ is released and H_2_ (or other gas species) is not influenced by the facilitated transport. A fixed carrier, which is bonded covalently to the polymer backbone, has limited mobility. Each CO_2_ molecule reacts with one carrier site and then passes to the next site until it reaches the permeate side [95,126].

Researchers have made attempts to establish FTMs in two directions: (i) development of innovative carriers that present high diffusivity and reactivity with CO_2_, and (ii) development of low-cost membranes that present very high selectivity. Stability in long-term operation is also necessary as membranes can be exposed to flue gases with NO_x_ and SO_2_ impurities. Until now, only a small number of polymeric membranes have shown to be efficient for CO_2_ capture, especially in pilot plants. Polyvinylamine (PVAm), which is a weak linear cationic polyelectrolyte that contains many primary amine groups, is widely reported as appropriate for facilitated CO_2_ transportation following various pathways with the presence of water [96,127]. Janakiram et al. (2019) [128] used an FTM that consists of a selective layer based on the blend of polyvinyl alcohol (PVA) with sterically hindered PVAm, and achieved 652 GPU of CO_2_ permeance, but a rather low CO_2_/N_2_ selectivity (41.3). In the following years, they scaled up and used three different hollow fiber FTMs with real flue gases from a cement plant [129] and simulated a two-stage membrane process using different classes of facilitated transport membranes, which were previously validated in industrial conditions, with promising results [130]. Wu et al. (2023) [66] also developed SW membrane modules for industrial use with an effective membrane area of 31 m^2^. The employed modules consisted of amine-based facilitated transport PVAm multilayer composite membranes, which were fabricated using roll-to-roll coating equipment. The results showed that a reduction in operating pressure increased the purity of CO_2_ but decreased the CO_2_ capture rate, and there was a trade-off between the power consumption and the membrane area demand. However, although PVAm-based modules have proven to be competitive, compared to most amine-based technologies, the difficulty in maintaining high performance in larger plants still prevents their full-scale application. Aiming to increase the mechanical strength of membranes, the main constituents of polymers coupled with other polymers or nanofillers, carbon nanotubes and metal–organic frameworks (MOFs) are also considered in FTMs [96].

## 6. Commercially Applied Membrane Modules for Industrial CO_2_ Capture

Three commercial membrane modules have been successfully demonstrated at a relatively high TRL (5–7) for post-combustion capturing of CO_2_ from flue gases of fossil fuel-fired and cement facilities: Polaris^TM^, PolyActive^TM^ and PRISM^TM^ modules [131].

Polaris^TM^, which was developed by Membrane Technology and Research (MTR) (USA), has been broadly examined and assessed for capturing CO_2_ from flue gases of coal-fired power plants. Polaris^TM^ Gen 1, which presents a CO_2_ permeance of 1000 GPU and a CO_2_/N_2_ selectivity of 50, has been tested commercially already [132,133,134]. During the years 2012–2015, Polaris^TM^ spiral-wound membranes exhibited prolonged membrane performance (>111,000 h) and achieved >90% CO_2_ capture on a bench scale (0.05 MWe or 1 tons of CO_2_/day). A larger pilot-scale system (1 MWe or 20 tons of CO_2_/day) also presented stable operation (>6 months), achieving 90% CO_2_ capture. In this unit, a plate-and-frame module with low-pressure drop, increased packing density and lower power cost (~10 MWe saving) was successfully operated as well. Polaris Gen 2, with twice the CO_2_ permeance but similar CO_2_/N_2_ selectivity, showed higher CO_2_ separation performance (60–70%), compared to Gen 1, and also exhibited stable performance for >40 h when it was tested on a laboratory scale. These efforts from MTR have advanced this membrane technology from TRL 2–3 to TRL 6. Aiming to capture 200 tons of CO_2_ per day from a coal-fired power plant, a large pilot system is under design with an expected CO_2_ purity *>* 99%. After the successful operation of such a plant, the commercial maturity of this membrane technology for CO_2_ capture is expected to increase to TRL 8. Finally, Polaris^TM^ membrane performance exhibited high stability (>168 h) during real field tests in 2021 at Jiangyou Power Plant (Jiangyou, China), which operated under dynamic conditions (power plant load ranged between 54 and 84%). The aforementioned results confirm the efficiency of Polaris^TM^ modules under varying operating conditions [32,135].

Helmholtz-Zentrum Geesthacht (Geesthacht, Germany) developed PolyActive^TM^ module, which showed high CO_2_ permeance, namely 3068 m^3^ (STP)/m^2^/h/bar (1136 GPU), and high CO_2_/N_2_ selectivity (60), with a CO_2_ recovery of 42.7% and a CO_2_ purity of 68.2% [32,136]. Nowadays, PolyActive™ membranes are broadly applied. Brinkman et al. [137] showed how temperature affects the transport properties of different multilayer PolyActive™ membranes; temperature increased permeance but decreased selectivity. At the optimal temperature (~40 °C), the permeance reached 2000 GPU and the CO_2_/N_2_ selectivity was almost 45. More information about the mechanical properties of PolyActive™ membranes (structure and thickness) is presented in the work by Schuldt et al. [138]. It was shown that when CO_2_ pressure is >8 bar, PolyActive™ swells with CO_2_, resulting in lower selectivity [13,138].

PRISM™ module, developed by the company Air Products (Allentown, PA, USA), was employed by Scholes et al. [139] at a Victorian brown coal-fired power plant. Initially, the pressure of the flue gas was increased to 150 kPa. The dried flue gas entered the membrane at 45 °C and 3.5 kg/h. After the blower discharge of a direct contact cooler, the module was placed under clean flue gas. However, water condensation on the membrane was the most important drawback of the process. Both selectivity and permeance were greatly reduced after a few hours of operation, which was attributed to membrane plasticization and swelling by water. After some time of operation, they increased slightly, but not up to the initial values. Apart from CO_2_, impurities of NO_x_ and SO_2_ also passed through the membrane. Scholes et al. [139] concluded that the main challenge was the fluctuations in humidity and the challenging regulation of the experimental setup in terms of temperature control. Due to humidity, significant changes can happen in membrane transport properties [13].

## 7. Main Challenges and Future Perspectives

**Figure 14:**
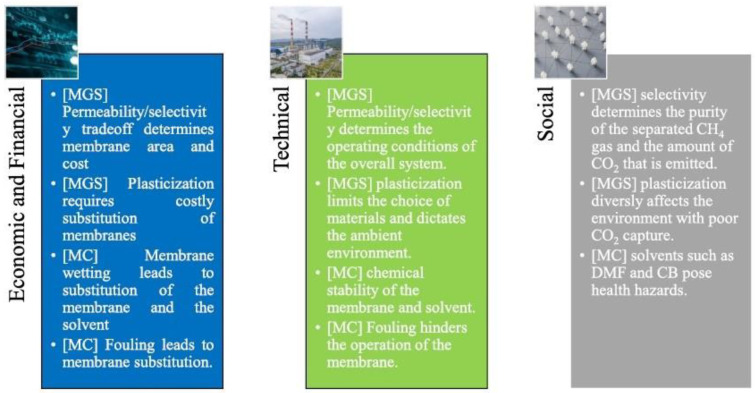
Economic, technical and social challenges for broad application of membrane-based CO_2_ capture (MGS: membrane gas separation, MC: membrane contactors) [143].

With the exception of some gas-sweetening membrane systems that are currently commercialized, the widespread application of membranes is still limited, and a significant gap is observed between lab-scale research and real field applications. Current commercial membrane modules present low selectivity, and multiple stages are needed to achieve the necessary CO_2_ purity [96,140]. Apart from the inevitable trade-off between selectivity and permeability, which still remains an important limitation in membrane separation technologies [14], a major challenge in coal-fired power plants is the low CO_2_ concentration (10–15%) in the emitted flue gases [141]. For these flue gases, commercial membranes cannot achieve the required CO_2_ selectivity and purity, illustrating the need for advanced, novel membranes [10]. Regarding the application of mixed-matrix membranes (MMMs) for CO_2_ capture, the main challenges include potential interfacial defects between the polymeric and inorganic phases, particle agglomeration, sedimentation and poor dispersion, which can decrease membrane selectivity. Apart from the size and amount of the employed particles, attention should be also given to chain rigidification, adhesion between particles and the polymer phase, viscosity of dope-containing particles and the applied stress to induce particle dispersion, which can influence the overall MMM performance [7,142]. The challenges that prevent the broad commercialization and application of membranes for carbon capture can be summarized into three categories, according to Olabi et al. (2023) [143]: economic/financial, technical and social (Figure 14).

Nevertheless, the number of membrane technologies for CO_2_ capture is expected to increase in the future in view of the benefits of low energy consumption, low capital and operating costs, low space requirements, avoidance of chemicals/harmful wastes and simple operation. Future research should focus on the improvement in separation process efficiency and the reduction in capital and operating costs, which will facilitate the use of membranes by interested stakeholders. To make membrane-based technologies cost-effective during carbon capture, further advancements are required in membrane fabrication with novel or composite materials (composed of two or more species), which will enhance the intrinsic and surface properties and, therefore, the selectivity and permeability of the produced membrane modules. It is also understood that the produced MMMs should be compatible with different flue gas compositions, apart from their increased selectivity. Although there are numerous studies concerning the use of MMMs for CO_2_ capture, industrial engineering is still in its infancy and the efforts to create new filler materials will be continued. Finally, the industrialization of MMMs for carbon capture will necessitate the utilization of low-cost, renewable and, if possible, naturally occurring polymers; the manufacturing and reproducibility of thin, defect-free bio-polymeric MMMs that will replace synthetic membranes is very challenging.

## 8. Conclusions

The most common post-combustion CO_2_ capture technologies are chemical absorption, adsorption, cryogenic separation and membrane separation. Membrane separation is increasingly implemented in recent years due to low capital and operating costs, low energy consumption and space requirements, easy operation, simple equipment, high flexibility, safety and an absence of possibly toxic or harmful wastes. The latest advancements in membrane technologies for CO_2_ capture emphasize primarily the fabrication of novel membranes with enhanced properties that will achieve higher selectivity, permeability, mechanical strength and potential for large-scale preparation. The majority of these membranes are composite or mixed-matrix membranes (MMMs), which are fabricated via the integration of novel inorganic materials (fillers) into the structure of polymeric membrane materials. Among these materials, recent progress focuses mainly on the utilization of metal–organic frameworks (MOFs), such as zeolitic imidazolate frameworks (ZIFs) and carbon molecular sieves (CMSs), i.e., activated carbons with molecule-sized pores. Other types of MMMs, which are increasingly examined for their potential to improve CO_2_ separation, include nanocomposite membranes, which employ nano-sized fillers of various materials; ionic liquid (IL)-based membranes, which employ ionic liquids as the continuous phase in the fabricated MMM; and facilitated transport membranes (FTMs), where the gas diffusion mechanism is based on facilitated transport.

## Figures and Tables

**Table 1:** Comparison of the main technologies for post-combustion CO_2_ capture [32].

*Technology*	*Mechanism*	*Advantages*	*Drawbacks*	*Maturity*
**Absorption**	Physical or chemical absorption of CO_2_ into a liquid carrier (solvent); regeneration via increase in temperature or reduction in pressure	High capture efficiency (>90%); aqueous amine scrubbing (MEA) is currently the benchmark carbon capture technology	Large energy penalty, estimated at 20–30% of the power plant output; solvent regeneration and CO_2_ recovery contributing ~50%; equipment corrosion and removal/disposal of solvent	*TRL 9*
**Adsorption**	Physical or chemical adsorption of CO_2_ using a solid sorbent; regeneration via increase in temperature or reduction in pressure	Lower regeneration energies compared to solvents due to lower heat capacities	Heat transfer, stability and attrition challenges	*TRL 7–9*
**Cryogenic separation**	Used for gas streams with high CO_2_ concentration (>90%)	Liquid CO_2_ produced is ready for transportation	Energy intensive	*TRL 9*
**Membrane separation**	Selective transportation and separation of CO_2_ through a membrane under the driving force of pressure difference	No hazardous chemicals storage, handling, disposal, or emissions issues; simple operation; reduced plant footprint; diminished need for modifications to the existing power plant steam cycle	Relatively low partial pressure of CO_2_ in the flue gas; use of low-cost and durable membranes; efficient permeability and selectivity; thermal, physical and chemical stability must be improved	*TRL 6*

**Table 2:** Performance of CO_2_/N_2_ separation for various polymer-based materials [61].

*Membrane Material*	*Permeance ^a^ (mol·s^−1^·m^−2^·Pa^−1^) or Permeability ^b^ (mol·s^−1^·m^−1^·Pa^−1^)*	*CO_2_/N_2_ Selectivity*	*Reference*
Cellulose acetate	2.48 × 10^−7 a^	40.17	[84]
Polyimides-TMeCat	6.30 × 10^−10 b^	25	[85]
Polyimides-TMMPD	1.89 × 10^−9 b^	17.1	[86]
Polyimides-IMDDM	6.17 × 10^−10 b^	18.1	[86]
Polysulfone-HFPSF-o-HBTMS	3.31 × 10^−10 b^	18.6	[87]
Polysulfone-HFPSF-TMS	3.47 × 10^−10 b^	18	[88]
Polysulfone-TMPSF-HBTMS	2.27 × 10^−10 b^	21.4	[89]
Polycarbonates-TMHFPC	3.50 × 10^−10 b^	15	[90]
Polycarbonates-FBPC	4.76 × 10^−11 b^	25.5	[91]

## Data Availability

No new data were created.
